# Phagocytic Activity Is Impaired in Type 2 Diabetes Mellitus and Increases after Metabolic Improvement

**DOI:** 10.1371/journal.pone.0023366

**Published:** 2011-08-18

**Authors:** Albert Lecube, Gisela Pachón, Jordi Petriz, Cristina Hernández, Rafael Simó

**Affiliations:** 1 Diabetes and Metabolism Research Unit, CIBER de Diabetes y Enfermedades Metabólicas Asociadas (CIBERDEM), Instituto de Salud Carlos III (ISCIII), Vall d'Hebron Institut de Recerca (VHIR), Hospital Universitari Vall d'Hebrón, Universitat Autònoma de Barcelona, Barcelona, Spain; 2 Research Unit in Biomedicine and Translational and Pediatrics Oncology, Vall d'Hebron Institut de Recerca (VHIR), Universitat Autònoma de Barcelona, Barcelona, Spain; Universita Magna-Graecia di Catanzaro, Italy

## Abstract

**Objective:**

1) To evaluate whether peripheral blood mononuclear cells (PBMCs) from type 2 diabetic patients present an impairment of phagocytic activity; 2) To determine whether the eventual impairment in phagocytic activity is related to glycemic control and can be reversed by improving blood glucose levels.

**Methods:**

21 type 2 diabetic patients and 21 healthy volunteers were prospectively recruited for a case-control study. In addition, those patients in whom HbA1c was higher than 8% (n = 12) were hospitalized in order to complete a 5-day intensification treatment of blood glucose. Phagocytic activity was assessed by using a modified flow cytometry procedure developed in our laboratory based on DNA/RNA viable staining to discriminate erythrocytes and debris. This method is simple, highly sensitive and reproducible and it takes advantage of classic methods that are widely used in flow cytometry.

**Results:**

Type 2 diabetic patients showed a lower percentage of activated macrophages in comparison with non-diabetic subjects (54.00±18.93 vs 68.53±12.77%; p = 0.006) Significant negative correlations between phagocytic activity and fasting glucose (r = −0.619, p = 0.004) and HbA1c (r = −0.506, p = 0.019) were detected. In addition, multiple linear regression analyses showed that either fasting plasma glucose or HbA1c were independently associated with phagocytic activity. Furthermore, in the subset of patients who underwent metabolic optimization a significant increase in phagocytic activity was observed (p = 0.029).

**Conclusions:**

Glycemic control is related to phagocytic activity in type 2 diabetes. Our results suggest that improvement in phagocytic activity can be added to the beneficial effects of metabolic optimization.

## Introduction

Patients with type 2 diabetes mellitus (T2DM) are immunocompromised and have an increased incidence of infections, mainly in the bone and skin, as well as in the respiratory, enteric and urinary tracts [1], [2]. In a Canadian retrospective cohort study that included 513,749 people with T2DM matched to an equal number of nondiabetic subjects the risk ratio for infectious disease-related hospitalization was 2.17 (99% CI 2.10–2.23) for the diabetic population, and the risk ratio for death attributable to infection was up 1.92 (1.79–2.05) [2]. Diabetic foot ulcers are often complicated by infection and represent the first cause of non-traumatic amputations and are a leading cause of hospitalization among diabetic patients [3]. Diabetes also influences the outcomes of specific infections, such as bacteremia and mortality following community-acquired pneumonia [1], [4]. In addition, it has recently been reported that diabetes triples the risk of hospitalization after influenza A (H1N1) infection and quadruples the risk of intensive care unit admission after hospitalization [5]. Furthermore, certain rare infections are more common among diabetic patients, including invasive otitis externa, rhinocerebral mucormycosis, and emphysematous infections of the gall bladder, kidney, and urinary bladder. Finally, postoperative hyperglycemia is an independent risk factor for surgical site infection [6]. Therefore, infections can be considered as a complication of diabetes and significantly contribute to its associated economic burden.

The reasons why diabetic patients present an increased susceptibility to frequent and protracted infections are still far from being understood. Several factors have been suggested, including genetic susceptibility to infection, local factors including poor blood supply and nerve damage, alterations in metabolism associated with diabetes, and defects in innate immune defence mechanisms [7]. Regarding the innate immunity, phagocytic activity of peripheral blood mononuclear cells (PBMCs) plays an essential role in protecting the host from infectious diseases, and decreased PBMC function has been implicated in the increased risk of infections that occurs in diabetic patients [8]. However, there are no specific studies addressed to evaluating whether impairment in phagocytic activity is related to glycemic control and, more importantly whether or not this impairment could be reversible after improving blood glucose levels.

To shed light to this issue we designed a case-control study comparing phagocytic activity between 21 type 2 diabetic patients (cases) and 21 healthy volunteers (controls) closely matched by age, gender and body mass index. The relationship between phagocytic activity and fasting glucose or HbA1c was determined. In addition, a subset of 12 type 2 diabetic patients who underwent 5-day blood glucose intensification treatment served to test the hypothesis that blood glucose optimization leads to a significant improvement in phagocytic activity. Finally, it is worthy of mention that phagocityc activity was assessed by using a modified flow cytometry procedure developed in our laboratory. This method is highly sensitive and reproducible, and takes advantage of classic methods widely used in flow cytometry [9].

## Materials and Methods

### Ethics statement

Informed written consent was obtained from all participants and the study was approved by the hospital's human ethics committee (Hospital Universitari Vall d'Hebron).

### Design of the study and description of study population

#### Case-control study

In this study we have investigated the effect of type 2 diabetes and the degree of glycaemic control on the phagocytic activity of monocytes/macrophages following the *Strengthening the Reporting of Observational Studies in Epidemiology* (STROBE) guidelines for reporting case–control studies [10].

We used the following formula for calculating the sample size:
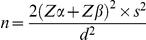
where the alpha level was set at p<0.05 (*Z*α) and the minimum acceptable power level was considered to be 0.80 (*Zβ*), and where *s* is the standard deviation in the mean percentage of phagocytic cells measured under basal conditions on healthy subjects, *d* is the postulated effect size (we considered clinically significant a difference in phagocytic activity between the two groups of 10%) and *n* is the sample size.
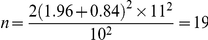



On this basis, a total of 21 consecutive type 2 diabetic patients of Caucasian origin and free of chronic diabetic complications who were attending the outpatient Diabetes Unit of a university hospital (Hospital Universitari Vall d'Hebron, Barcelona, Spain) were recruited for the study over a 2-month period (cases). We aimed to select one control for every case and, consequently, 21 non-diabetic healthy volunteers served as a control group. Controls were individually matched to cases by age, gender, and BMI.

#### Interventional study

The 12 type 2 diabetes patients with the poorest glycemic control (HbA1c>8%) among the 21 patients recruited for the case-control study agreed to participate in the interventional study. In this study phagocytic activity was determined at baseline and 5-days after improving blood glucose control.

Six of the 12 patients were under combined treatment with metformin plus insulin once a day. In these patients short acting insulin was added before breakfast, lunch and dinner. Four patients were under metformin plus sulphonylureas. In these patients metformin was maintained and suphonylureas were replaced by long acting insulin. The remaining two patients were under metformin treatment alone and were optimized by diet.

In order to minimize the variables that could influence PBMCs function patients were admitted to our Diabetes Unit for a short period (5 days) and they were on the same diet.

The exclusion criteria for both studies included active infection, malignancy, chronic or acute renal failure, smoking habit, chronic respiratory disease, heart failure, anti-inflammatory or immunosuppressive treatment, and chronic diabetic complications. A complete physical examination and chest radiography were performed on all patients included in the study.

Type 2 diabetes was defined according to the criteria recommended by the Expert Committee on the Diagnosis and Classification of Diabetes. On this basis, all type 2 diabetic cases were diagnosed by two fasting plasma glucose values equal or higher than 7.0 mmol/l.

### Measurement of phagocytic activity and flow cytometric analysis

After an overnight fast of 10 h, venous blood was collected from the antecubital vein in preheparinised syringes at 8.00 am. During the next 60 minutes 100 µL of the heparinized whole blood was exposed for 30 minutes at 37°C in the dark to 20 µL of the commercial pHrodo™ *E. coli* BioParticles® phagocytosis kit for flow cytometry (Invitrogen, Eugene, Oregon, USA). Another sample was placed at 4°C in order to prevent active phagocytosis. Fluorescence activity at 4°C provided a measure of the nonspecific binding of *E. coli* with the granulocytes. Finally, cells were resuspended in 400 µL of Hank's BSS (1X) without Ca^2+^ and Mg^+^ and without phenol red (PAA, Austria) solution, and 20 µL of Hoechst 33342 (Invitrogen, Eugene, Oregon, USA) at 5 µg/10^6^ cells labelling (final concentration of 40 µg/mL) was added to discriminate the nucleated cells. The samples were ready for analysis using a MoFlo® flow cytometer (Beckman-Coulter, Hialeah, FL) equipped with 351 and 488 nm gas lasers. The nucleated phagocytes were discriminated from non nucleated cells and debris by gating on the granulocyte and monocyte populations using forward and scatter properties. During data acquisition, a ‘live’ gate was set in the blue fluorescence vs. side scatter dotplot on those events (Hoechst blue fluorescence; BP filter of 405 nm). For phagocytosis assays, nucleated cells were labelled simultaneously with Hoechst 33342 and pHrodo™ *E. coli* BioParticles®. A side scatter vs. Ho342 dotplot was used to discriminate nucleated cells from erythrocytes and debris. Threshold was set in forward scatter. Phagocytic cells were then selected in a forward vs. side scatter dotplot. Cell subpopulations with different phagocytic levels were measured using a side scatter vs. pHrodo™ *E. coli* BioParticles® contour plot.

### Laboratory assessment of metabolic parameters

Fasting plasma glucose, fructosamine and HbA1c were measured from the same blood sample used to isolate granulocyte and monocyte activity. FPG was determined by the hexoquinase method (Olympus Diagnostica GmbH, Hamburg, Germany) and fructosamine was determined using a colorimetric method (Sentinel CH, Milan, Italy). Finally, HbA1c was determined by chromatography. During the 5-day inpatient period, a nine-point capillary blood glucose profile was performed on days 1, 3 and 5.

### Statistical analysis

Normal distribution of the variables was evaluated using the Kolmogorov–Smirnov test. Data were expressed as the means and standard deviation. Data from the flow cytometer were analyzed using FlowJo Software (Tree Star Inc.) and were expressed as percentages (mean ± SD). Comparisons between groups were made using the Student's *t* test for continuous variables and the χ^2^ test for categorical variables. The relationship between the continuous variables was examined by the Spearman's rank correlations. In addition, stepwise multiple linear regression analyses were performed. The percentage of active phagocytic cells was considered as the dependent variable, and the independent variables were: age, gender, and BMI, along with the variables associated with phagocytosis activity in univariate analysis (fasting plasma glucose and HbA1c).

All p values were based on a two-sided test of statistical significance. Significance was accepted at the level of p<0.05. Statistical analyses were performed using the SSPS statistical package (SPSS, Chicago, IL, USA).

## Results

### Case-control study

**Figure 1:**
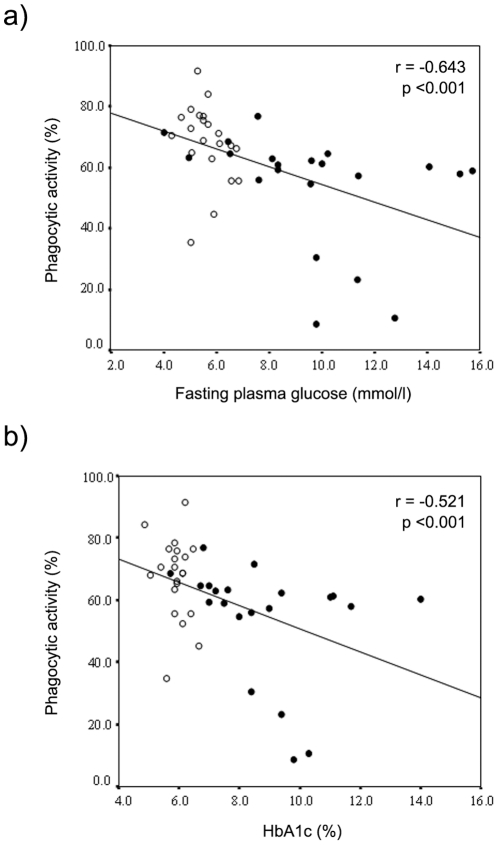
Correlations of fasting plasma glucose (a) and HbA1c (b) with phagocytic activity in the whole population. White circles, non-diabetic patients (fasting glucose: r = −0.419, p = 0.058; HbA1c: r = −0.011, p = 0.956); black circles, type 2 diabetic patients (fasting glucose: r = −0.601, p = 0.004; HbA1c: r = −0.506, p = 0.019).

The main clinical features of the study population are shown in Table 1. Diabetic patients showed a lower percentage of active phagocytic cells (54.00±18.93 *vs.* 68.53±12.77%; mean difference 14.53 [95% CI −24.64 to −4.41]; p = 0.006) in comparison with non-diabetic patients. Univariate analysis showed that in the whole population, as well as in diabetic patients, phagocytosis activity negatively correlated with fasting plasma glucose (r = −0.643, p<0.001 and r = −0.601, p = 0.004; respectively) and HbA1c (r = −0.521, p<0.001 and r = −0.506, p = 0.019; respectively) (Figure 1). By contrast, we did not observe any correlation between the percentage of activated macrophages and neutrophils and either age (r = −0.180, p = 0.254) or BMI (r = 0.221, p = 0.160). Stepwise multiple linear regression analysis showed that fasting plasma glucose (beta = −0.475, p = 0.001), was independently associated with phagocytosis activity (R^2^ = 0.326).

**Table 1:** Main clinical characteristics and metabolic data of participants included in the case-control study according to the presence of type 2 diabetes.

	T2DM(n = 21)	Control group(n = 21)	Mean difference(95% CI)	p value
**Age (yrs)**	54.95±8.22	51.57±11.85	3.38 (−2.98 to 9.74)	0.290
**Women, n (%)**	13 (61.90)	17 (80.95)	-	0.153
**BMI (Kg/m^2^)**	36.44±6.93	37.29±9.81	−0.85 (−6.15 to 4.45)	0.748
**Fasting glucose (mmol/l)**	9.49±3.15	5.73±0.83	3.76 (2.25 to 5.27)	<0.001
**HbA1c (%)**	8.78±2.01	5.79±0.45	2.99 (2.05 to 3.92)	<0.001
**Phagocytosis (%)**	54.00±18.93	68.53±12.77	−14.53 (−24.64 to −4.41)	0.006

### Interventional study

**Figure 2:**
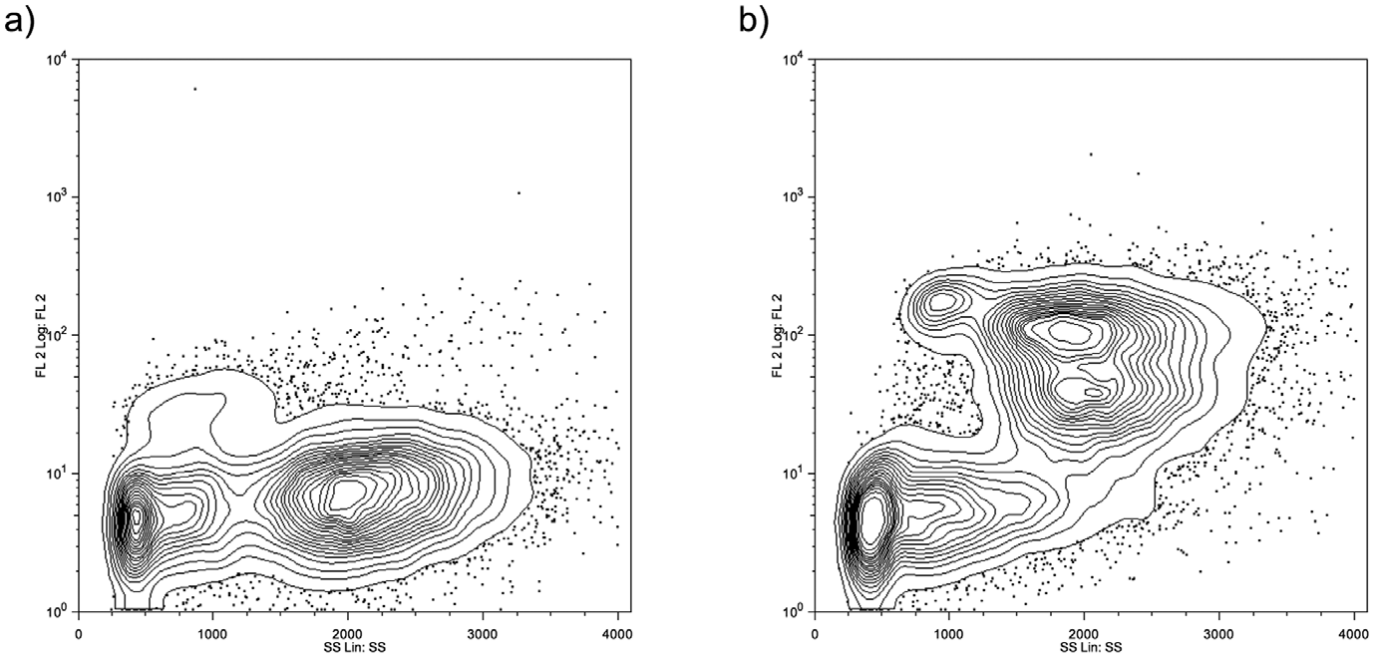
Flow cytometry graphic measuring phagocytic activity in a representative case of type 2 diabetic patient before (a) and after (b) 5-day intensification treatment of blood glucose.

During the 5-day intensification treatment of blood glucose, a significant reduction in 9 point glycemic profile, fructosamine and HbA1c was achieved (table 2). This improvement in metabolic control was associated with a significant increase in the percentage of activated PMBCs (64.52±14.67 at day 1 *vs.* 78.82±18.74% at day 5; mean difference −14.30 [95% CI −26.81 to −1.80], p = 0.029) (Table 2). Notably, an improvement in phagocytic activity was observed in 9 out of 12 of T2DM patients. The flow cytometry graphic measuring phagocytic activity before and after blood glucose normalization in a representative case is displayed in Figure 2.

**Table 2:** Main clinical characteristics and metabolic data at baseline (day 1) and discharge (day 5) of type 2 diabetic patients (n = 12) included in the interventional study.

	Day 1	Day 5	Mean difference(95% CI)	p value
**Age (yrs)**	52.25±13.40	-	-	-
**Women, n (%)**	7 (58.33)	-	-	-
**BMI (Kg/m^2^)**	30.77±6.85	30.58±6.42	0.18 (−0.26 to 0.63)	0.381
**9-point glucose profile**	11.41±2.83	7.71±1.51	3. 70(1.18 to 6.22)	0.009
**HbA1c (%)**	9.03±2.04	8.71±2.05	0.31 (0.13 to 0.50)	0.003
**Fructosamine (mg/dl)**	395.73±64.62	377.91±54.62	17.82 (4.72 to 30.92)	0.013
**Phagocytosis (%)**	64.52±14.67	78.82±18.74	−14.30 (−26.81 to −1.80)	0.029

## Discussion

In the present study we provide evidence that in T2DM patients the attenuated phagocytic activity of PMBCs exhibits a significant reversibility after glycemic control is improved. In addition, our baseline results confirm and extend previous reports concerning the impairment of phagocytosis that occurs in PMBCs from diabetic patients. Finally, our findings suggest that blood glucose control plays an essential role in accounting for the low phagocytic activity detected in T2DM patients.

Previous studies have described diminished PMBC function (i.e. defective chemotaxis, bacterial killing, superoxide production, leukotriene release, lysosomal-enzime secretion and endoplasmic reticulum stress) as well as altered basal levels of intracellular calcium and superoxide in diabetes [11]–[16]. However, in most of these investigations the degree of glycemic control was not reported.

Given the complex network of interacting cells and mediators that collectively contribute to both innate and acquired immunity, we have focused our study on phagocytic activity in T2DM taking into account the degree of metabolic control. Our results agree with previous studies showing an impairment of phagocytic activity of PBMCs in subjects with T2DM [8], [11], [14]–[17]. However, a normal or even enhanced functional responsiveness of diabetic-subjects' PBMCs has also been reported [18], [19]. These controversial results are in part due to the heterogeneity of diabetic subjects, insufficient numbers in the study population, the lack of a closely matched control group, inconsistency in the collection of the cell population under investigation, and different methods for measuring phagocytic activity. In the present study a homogenous population of patients with T2DM was analyzed, the number of subjects included was sufficient for the purpose of the study, a close matched control group was included, the collection of PMBCs was accurate and the method used for measuring phagocytic activity was highly sensitive and reproducible. In this regard it should be emphasized that this method is based on DNA/RNA viable staining to discriminate erythrocytes and debris and avoids lysis, and the washing and centrifugation steps, thus making it more reliable than classic methods of flow cytometry for measuring phagocytic activity [9]. Nevertheless, further studies addressed to examining the clinical usefulness of this method for evaluating the risk of diabetic patients to acquire infections are needed.

Phagocytic cells are the cornerstone of the innate immune system, and individuals with inherited defects in phagocyte function typically present recurrent, unusual, and/or difficult to clear bacterial infections [20]. Our findings reinforce the theory that, in contrast to the sustained immune deficits that occur in genetic disorders, the extent of impairment of phagocytosis in diabetic patients oscillates in relation to glycemic control. Therefore, persistently poor glycemic control could have a progressively deleterious effect, predisposing affected individuals to an increased incidence and severity of infection. In addition, our results suggest that an improvement of phagocytic activity can be considered as another beneficial effect of blood glucose optimization.

The reasons why hyperglycemia or related pathways downregulate phagocytic activity remain to be elucidated. In vitro data demonstrate that the response of leukocytes stimulated with inflammatory mediators is inversely correlated with indices of in vivo glycemic control [15]. In addition, endoplasmic reticulum stress has been found in PBMCs in patients with diabetes [16]. The deleterious effects of free radicals are enhanced in diabetes, because they are less easily eliminated as a result of the reduced effectiveness of the protecting agents. Diabetes activates aldose-reductase which leads to a reduction of glucose excess in sorbitol (polyol pathway). This pathway is a great consumer of NADPH, which is then less available for PBMC functions and for free radical elimination by antioxidants. In fact, aldose reductase inhibition enhances neutrophil oxidative killing by diabetic neutrophils [21]. Finally, mithocondrial dysfunction could be involved in the phagocytic impairment detected in diabetic patients [22].

The reversibility of phagocytosis dysfunction after a short period of glycemic control may have been attributable not only to the normalization of the diabetic milieu but also to the effect of exogenous insulin. However, it should be noted that in those patients who were under treatment with insulin, optimization was achieved without a significant increase in total insulin dosage. In addition, a significant improvement of phagocytic activity was observed in the two patients in whom optimization was achieved by diet alone. These findings point to glycemic optimization (and the normalization of the metabolic pathways triggered by hyperglycemia) rather than the effect of insulin *per se* as the main factor accounting for the improvement of phagocytic function. Of the twelve patients included in the interventional study, three did not improve phagocytic activity. However, in comparison with the nine patients whose their phagocytic activity increased, there were no differences relating to age, gender, baseline phagocytic activity, degree of metabolic control and anthropometric measures. In addition, no differences in response to therapeutical intervention were observed.

Finally, most of our patients were obese (BMI>30 kg/m^2^). Epidemiological data show that as occurs in diabetes, obese patients are more likely to acquire infections [23]. When analyzed by flow cytometry, obese subjects appear to have an altered frequency of circulating T lymphocytes compared with lean controls, as well as a lowered capacity of lymphocytes to respond to mitogen stimulation [24], [25]. Molecular factors linking adipose tissue and immunity processes have been described. In this way, GATA2, a transcription factor specifically expressed in the stromal vascular fraction of the adipose tissue, blocks preadipocyte-adipocyte differentiation and is involved in the inhibition of macrophage activation [26]. Menghini *et al* provided evidence that when GATA2 is blocked through its phosphorylation on Ser401, preadipocytes convert to adipocytes at the time that a significantly attenuation in their phagocytic activity is observed [27]. Therefore, obese subjects with T2DM are especially liable to present abnormalities in phagocytic function and, therefore, represent a target population for improving phagocytic function by blood glucose optimization.

Although our data on the improvement of phagocytosis dysfunction after a short period of glycemic control might also be transferable to long-term glucose lowering treatment, we have no data on this issue and, therefore, this is a limiting factor of our study.

In conclusion, the presence of T2DM and the degree of glycaemic control are related to substantially impaired phagocytosis activity which is reversible after glycemic control has been improved. Our results suggest that an improvement in phagocytic activity can be added to the beneficial effects of metabolic optimization. Further studies addressed to examining whether this improvement of phagocytic activity leads to a reduction of the rate of infection in diabetic patients are needed.
